# Role of Plasma Membrane NADPH Oxidase in Response to Salt Stress in Cucumber Seedlings

**DOI:** 10.3390/antiox11081534

**Published:** 2022-08-06

**Authors:** Katarzyna Kabała, Małgorzata Reda, Anna Wdowikowska, Małgorzata Janicka

**Affiliations:** Department of Plant Molecular Physiology, Faculty of Biological Sciences, University of Wrocław, Kanonia 6/8, 50-328 Wrocław, Poland

**Keywords:** ascorbate peroxidase, catalase, hydrogen peroxide, NADP dehydrogenases, NADPH oxidase, salt stress, superoxide dismutase

## Abstract

Plasma membrane NADPH oxidases (RBOHs, EC 1.6.3.1) are known as the main ROS generators involved in plant adaptation to stress conditions. In the present work, regulation of NADPH oxidase was analyzed in cucumber (*Cucumis sativus* L. var. Krak) seedlings exposed to salinity. RBOH activity and gene expression, as well as H_2_O_2_ content, were determined in the roots of plants treated with 50 or 100 mM NaCl for 1 h, and 50 mM NaCl for 1 or 6 days. It was found that enzyme activity increased in parallel with an enhancement in the H_2_O_2_ level in roots exposed to 100 mM NaCl for 1 h, and to 50 mM NaCl for 1 day. The expression of some *CsRboh* genes was induced by salt. Moreover, an increase in the activity of G6PDH, providing the substrate for the NADPH oxidase, was observed. In seedlings subjected to salinity for a longer time, antioxidant enzymes—including superoxide dismutase, catalase, and ascorbate peroxidase—were activated, participating in maintaining a steady-state H_2_O_2_ content in the root cells. In conclusion, NADPH oxidase and endogenous H_2_O_2_ up-regulation seem to be early events in cucumber response to salinity.

## 1. Introduction

In plants, reactive oxygen species (ROS), including the superoxide radical (O_2_^−^) and hydrogen peroxide (H_2_O_2_), are known to act as harmful compounds responsible for the oxidation and damage of numerous cellular components. On the other hand, when present in low concentrations, they are essential signaling molecules involved in the regulation of multiple physiological processes that affect plant growth and development [1]. ROS production may occur through both enzymatic and non-enzymatic pathways, among which plasma membrane NADPH oxidases (respiratory burst oxidase homologs, RBOHs) are the most studied of enzymes considered to be the main ROS generators [2]. Both ROS and RBOHs have been shown to participate in cell growth via regulation of cell wall loosening and stiffening. RBOH-dependent spatial and temporal control of ROS production is necessary for proper cell elongation. It has been demonstrated that *Arabidopsis* and barley *Rboh* mutants display impaired growth [3].

RBOHs are integral membrane proteins containing several functional domains, including haem cofactors, FAD, and calcium-binding (EF-hand) motifs. They catalyze an electron transfer from cytosolic donor NADPH to extracellular acceptor O_2_ via FAD and two haems, forming O_2_^−^, followed by its dismutation to H_2_O_2_ [4]. Studies using *Arabidopsis* have revealed that NADPH oxidase activity may be regulated at post-translational level synergistically by phosphorylation and Ca^2+^ binding [5]. More recently, it has been indicated that NADPH oxidases remain under the control of differential cellular effectors, such as small GTPases, calcium sensors CBL-CIPKs (calcineurin B-like protein-CBL-interacting protein kinases), and phosphatidic acid, the lipid product of phospholipase D [2]. Plant NADPH oxidases are encoded by small multigenic families. Ten *Rboh* genes have been identified in *Arabidopsis*, whereas nine isogenes are present in the genomes of rice and cucumber [3,6]. Differential *Rboh* expression, both in plant organs/tissues and at distinct developmental stages, suggests specific or housekeeping functions for individual RBOH isoforms [3].

In addition to the plasma membrane NADPH oxidases, apoplastic H_2_O_2_ generation can also be mediated by oxidative degradation of polyamines (PAs) [7]. The cell wall PA-catabolizing amine oxidases are classified into copper-containing diamine oxidases (DAOs, also known as CuAOs) and flavin-dependent polyamine oxidases (PAOs). DAOs oxidize mainly putrescine, whereas the apoplastic PAOs terminally degrade spermidine and spermine, both yielding aminoaldehydes and H_2_O_2_ [7]. Consequently, an increase in the extracellular H_2_O_2_ pool appears. It has been suggested that H_2_O_2_ generated as a product of PA oxidation may be directly involved in cell signaling processes, as well as in plant adaptation to abiotic stresses [8]. It is worth noting that PAO is highly expressed mainly in monocots, whereas DAO is active at high levels in dicotyledons [8].

In their natural environment, plants are exposed to unfavorable factors, including drought, salt, and extreme temperatures, which are perceived by cells. Tolerance of plants to salinity is governed by numerous physiological and molecular adaptive mechanisms [9]. Some of them involve ROS signaling [3,9]. Several studies have suggested that the plasma membrane NADPH oxidase may play an important role in plant acclimation to salinity, participating in both transcriptional and post-translational regulation. It has been shown, that under salinity, NADPH oxidase activity and endogenous ROS production are involved in the elevation of Ca^2+^ levels [10], the stabilization of the plasma membrane Na^+^/H^+^ antiporter, AtSOS1, mRNA [11], the regulation of Na^+^/K^+^ homeostasis via activation of plasma membrane Ca^2+^-permeable channels as well as inwardly rectifying K^+^ channels [12], and NaCl-induced activation of antioxidant enzymes [13].

Other studies have revealed that salinity induces the PA catabolism pathway into the apoplast, generating H_2_O_2_, and thereby activating tolerance mechanisms [7]. Tanou, et al. [14], observed that PAO and DAO encoding genes were remarkably induced by salinity treatments. They showed that PA degradation by DAO and PAO represents an active source for H_2_O_2_ generation. H_2_O_2_ produced by PA catabolism may activate antioxidative defense responses [14]. More recently, the interplay between amine oxidases and NADPH oxidases was examined in tobacco subjected to salt stress [15]. RBOH and apoplastic PAO were found to cooperate in controlling the accumulation of H_2_O_2_ and superoxides O_2_^−^. Both enzymes are a part of the same ROS regulatory network, in which NADPH seems to act upstream of apoplastic PAO [15].

The aim of the present study was to understand the role of the plasma membrane NADPH oxidase in the regulation of the H_2_O_2_ level, and its interrelation with antioxidant enzymes (superoxide dismutase, catalase, and ascorbate peroxidase) in the roots of cucumber seedlings exposed to salinity. Roots are the first organ exposed to unfavorable factors that get into plants from the soil. It was expected that under conditions of different time-exposure to salt, the plasma membrane NADPH oxidase activity would be modulated in a manner dependent on its gene expression and substrate (NADPH) delivery, and that, consequently, the content of the H_2_O_2_ would be modified. Moreover, it was assumed that changes in NADPH oxidase activity are related to the action of antioxidant enzymes.

## 2. Materials and Methods

Cucumber (*Cucumis sativus* L. var. Krak) seeds (originating from W. Legutko, Jutrosin, Poland) were germinated in darkness at 25 °C for 2 days. Seedlings were grown in containers (one container for each treatment) filled with nutrient solution (pH 6.5) consisting of 1.7 mM KNO_3_, 1.7 mM Ca(NO_3_)_2_·4H_2_O, 0.33 mM KH_2_PO_4_, 0.33 mM MgSO_4_·7H_2_O, and micronutrients: 75 μM ferric citrate, 10 μM MnSO_4_·5H_2_O, 5 μM H_3_BO_4_, 1 μM CuSO_4_·5H_2_O, 0.01 μM ZnSO_4_·7H_2_O, 0.05 μM Na_2_MoO_4_·2H_2_O without (control) or with the addition of salt. The plants were treated with 50 or 100 mM NaCl for 1 h immediately before analysis (50/1H and 100/1H, respectively), 50 mM NaCl for 1 day (50/1D; the seedlings were grown for 5 days without NaCl, and then transferred to an NaCl-containing medium for the next 24 h) or 50 mM NaCl for 6 days (50/6D; the seedlings were grown for 6 days in an NaCl-containing medium). Concentration of 5 mM H_2_O_2_ was added to the control nutrient solution for 24 h. The plants were grown hydroponically under a 16-h photoperiod at temperatures of 25 °C/22 °C (day/night), photon flux density 180 mol m^−2^s^−1^, and about 70% relative humidity. Whole roots were collected from all the cucumber plants in the container (200 plants/1 dm^3^ or 20 plants/100 cm^3^ of nutrient solution, depending on the biochemical measurement), and used for analysis.

H_2_O_2_ was quantified according to the method of Velikova, et al. [16], with some modifications as described by Kabała, et al. [17], based on the oxidation of potassium iodide (KI). After incubation in darkness at room temperature for 60 min, the absorbance of the reaction product, triiodide (I_3_^−^), was measured at 390 nm.

The plasma membrane fractions were isolated from the cucumber roots in accordance with the method of Larsson [18], as modified by Kłobus [19], using a 6.2% two-phase system containing PEG (polyethylene glycol) 3350 and dextran T500. The upper phase was enriched in highly purified plasma membrane vesicles.

The catalytic activity of the NADPH oxidase (EC 1.6.3.1) was determined in the plasma membrane fractions, according to the modified method of Sagi and Fluhr [20], as described by Jakubowska, et al. [6], using 0.5 mM XTT (2,3-bis(2-methoxy-4-nitro-5-sulfophenyl)-2H-tetrazolium-5-carboxanilide inner salt) and 0.1 mM NADPH as an electron donor. XTT reduction by an NADPH oxidase-generated O_2_^−^ radical was determined at 470 nm in the presence and absence of 50 units of CuZn-SOD. NADPH oxidase in-gel assays were performed according to the method described by Sagi and Fluhr [20]. The plasma membrane fractions were subjected to Native PAGE, using 7.5% (*w*/*v*) polyacrylamide gel. Electrophoresis was conducted at 110 V and 20 mA for 60 min. The NADPH-dependent O_2_^−^ production was assayed in gel by a modified NBT (nitroblue tetrazolium) reduction method. After the NADPH addition, the appearance of blue formazan bands was monitored.

Diamine oxidase (DAO, EC 1.4.3.6) activity was assayed in seedling roots spectrophotometrically, using putrescine as a substrate, in accordance with the method of Holmsted, et al. [21], as modified by Federico and Angelini [22]. During the reaction, Δ-pyrroline, formed by the enzymatic oxidation of putrescine, reacted with 2-aminobenzaldehyde to produce a yellowish-colored dihydroquinazolinium derivative. The absorbance was measured at 430 nm (e = 1.86 10^3^ mol^−1^ cm^−1^).

The activities of enzymes generating NADPH, 6PGDH (6-phosphogluconate dehydrogenase, EC 1.1.1.44), G6PDH (glucose-6-phosphate dehydrogenase, EC 1.1.1.49), NADP-ICDH (NADP-isocitrate dehydrogenase, EC 1.1.1.42), and NADP-ME (NADP-malic enzyme, EC. 1.1.1.40), were determined in the cucumber roots in accordance with the method of Li, et al. [23], as described by Jakubowska, et al. [6]. Generation of the NADPH was monitored spectrophotometrically by measuring the reduction of the NADP at 340 nm.

Catalase (CAT, EC 1.11.1.6) activity was determined according to the method of Aebi [24], as described by Janicka-Russak, et al. [25]. H_2_O_2_ decomposition was monitored spectrophotometrically by measuring the reduction in absorbance at 240 nm. One unit of CAT is defined as the amount of enzyme that breaks down 1 µmol of H_2_O_2_ per min.

Ascorbate peroxidase (APX, EC 1.11.1.11) activity was determined in accordance with the method of Chen and Asada [26], as described by Janicka-Russak et al. [25]. The H_2_O_2_-dependent oxidation of ascorbic acid was followed by monitoring the decrease of absorbance at 290 nm, assuming an absorption coefficient value of 2.8 mM^−1^ cm^−1^. One unit of APX is defined as the amount of enzyme that oxidizes 1 µmol of ascorbic acid per min.

Superoxide dismutase (SOD, EC 1.15.1.1) activity was measured in accordance with the method (xanthine/xanthine oxidase test) of Beauchamp and Fridovich [27]. The photoreduction of NBT and the formation of blue formazan was measured at 560 nm; an inhibition curve was made against different volumes of extract (5–20 µL). One unit of SOD is defined as the enzyme activity that inhibits the reduction of NBT to blue formazan by 50%.

Protein content was determined according to the method of Bradford [28], using bovine serum albumin (BSA) as the standard.

The total RNA was isolated from the cucumber roots using Tri Reagent (Sigma, St. Louis, MO, USA), and cDNA was synthetized with a High-Capacity cDNA Reverse Transcription Kit (Applied Biosystems, Waltham, Massachusetts, USA), in accordance with the manufacturer’s instructions, as described by Jakubowska, et al. [6].

The expression profile of the NADPH oxidase and SOD encoding genes in the cucumber roots was analyzed with the LightCycler 480 system (Roche, Basel, Switzerland). The cDNA was used as a template in a real-time PCR reaction with the Real-Time 2× PCR Master Mix SYBR kit (A&A Biotechnology, Gdańsk, Poland), performed in accordance with the manufacturer’s instructions, as described by Jakubowska, et al. [6]. The amplification conditions were as follow: 30 s at 95 °C; 40–45 cycles of 10 s at 95 °C; 10 s at 58 °C (for SOD genes) and 57 or 59 °C (for RBOH genes); 12 s at 72 °C; and final melting for 15 s at 65 °C. Genes encoding the cucumber tonoplast intrinsic protein, *CsTIP41*, and the clathrin adaptor complex subunit, *CsCACS*, were used as the internal standards [29]. Primer sequences specific to amplified genes, designed by LightCycler ProbeDesign software (Roche, Basel, Switzerland), are listed in Table 1. A melting curve analysis was performed to confirm the specificity of amplification and the absence of nonspecific by-products.

All data presented are expressed as the means of at least three biological replicates (each performed with two or three analytical replications) ± standard deviation (SD). One-way ANOVA and Duncan’s post hoc analysis (significant at *p* < 0.05) were used for statistical analysis, performed with Statistica 13.3 (TIBCO Software Inc., Palo Alto, CA, USA; 2017).

## 3. Results

**Figure 1:**
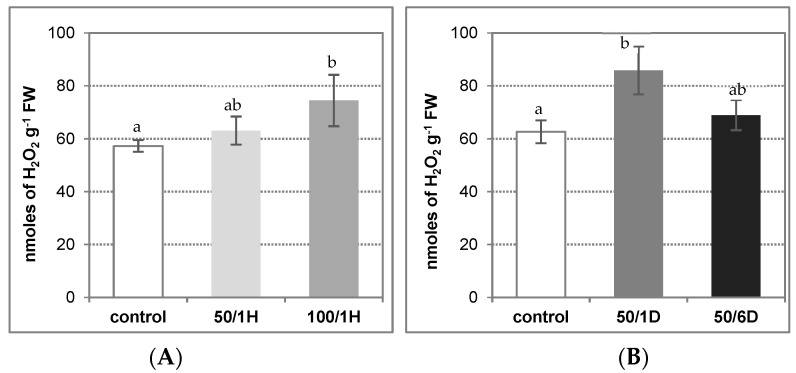
H_2_O_2_ level in roots of cucumber seedlings treated with: (**A**) 50 mM or 100 mM NaCl for 1 h (50/1H and 100/1H, respectively); (**B**) 50 mM NaCl for 1 or 6 days (50/1D and 50/6D, respectively). The H_2_O_2_ level was determined in the roots as described in Materials and Methods. The presented results are the means ± SD of three independent experiments, each run in triplicate. Different letters show different homogeneous groups of means calculated by Duncan’s multiple range test (significant at *p* < 0.05).

Environmental stress factors are known to elevate endogenous levels of small signaling molecules, including hydrogen peroxide, in plant tissues. Accordingly, H_2_O_2_ content was measured in the roots of cucumber seedlings exposed to 50 or 100 mM NaCl for 1 hour, and to 50 mM NaCl for 1 or 6 days. It was observed that 1-h-treatment of plants with 100 mM salt resulted in a visible increase in the H_2_O_2_ level, whereas 50 mM salt had no significant effect. The H_2_O_2_ achieved 130% and 110%, respectively, of the control (Figure 1A). As a result of longer treatment of seedlings with 50 mM NaCl concentration, the H_2_O_2_ content increased, reaching about 137% of the control value after 24 h, and then decreased in 6-day exposed plants, to the level observed in 1-h-treated seedlings, i.e., 110% of the control (Figure 1B).

**Figure 2:**
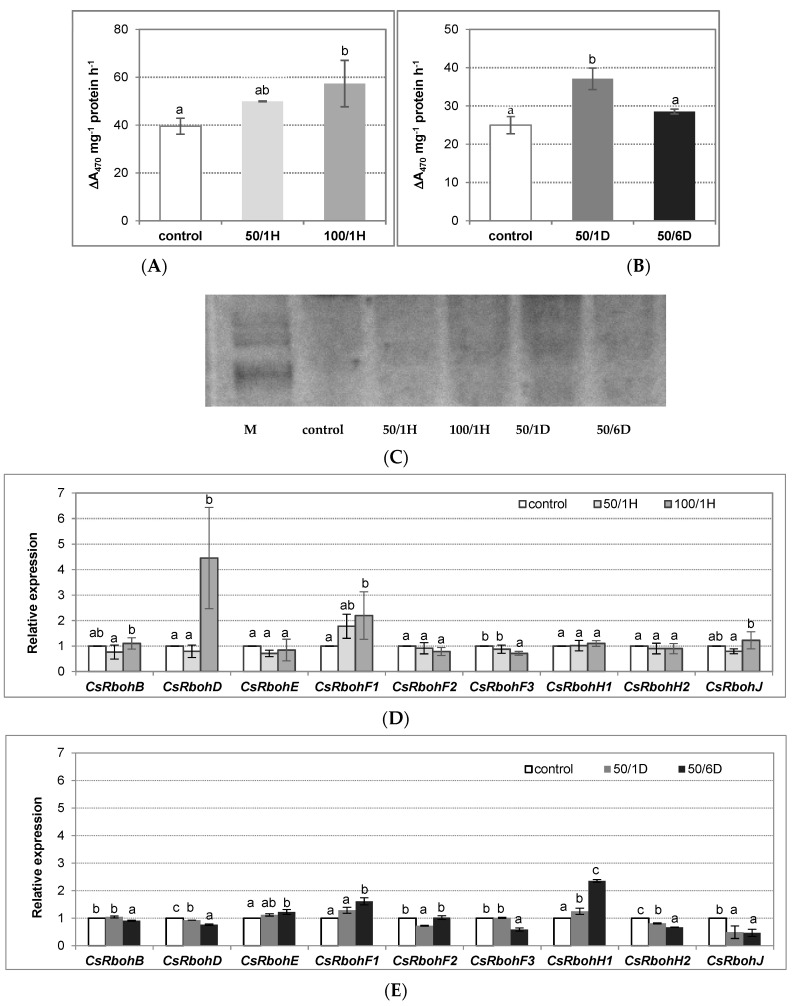
NADPH oxidase catalytic activity (**A**,**B**), in-gel activity(**C**), and relative expression of *CsRboh* genes (**D**,**E**) in the tissues of cucumber roots exposed to salinity. The cucumber seedlings were treated with: (**A**,**C**,**D**) 50 mM or 100 mM NaCl for 1 h (50/1H and 100/1H, respectively) and (**B**,**C**,**E**) 50 mM NaCl for 1 or 6 days (50/1D and 50/6D, respectively). The plasma membrane vesicles were isolated from the cucumber roots to determine both catalytic (**A**,**B**) and in-gel (**C**) NADPH oxidase activity, as described in Materials and Methods. (**A**,**B**) The presented results are the means ± SD of three independent experiments, with each experiment performed in triplicate, and are expressed as the ΔA_470_ of formazan XTT mg^−1^ protein h^−1^. (**C**) Gel image with blue formazan bands detected using the NBT method. The plasma membrane proteins were subjected to native PAGE, and examined for their ability to generate O_2_^−^ by reduction of nitroblue tetrazolium. The bands detected in the gel were due to NADPH-dependent NBT reduction by O_2_^−^ radicals. The gel image shown is representative of three independent replications of the experiment; M—molecular weight marker. (**D**,**E**) The total RNA was isolated from the cucumber roots, and real-time PCR analysis was performed with *CsTIP41* as a reference gene, as described in Materials and Methods. The results are shown as means ± SD of three replications. Different letters show different homogeneous groups of means calculated by Duncan’s multiple range test (significant at *p* < 0.05).

The plant NADPH oxidases are the main enzymes that participate in H_2_O_2_ production. Using two methods, we demonstrated that the treatment of seedlings with NaCl stimulated NADPH oxidase activity in the roots. The activity achieved about 126% and 145% of the control level in plants exposed to 50 mM and 100 mM NaCl, respectively, for 1 h (Figure 2A,C). When plants were treated with 50 mM NaCl for a longer time, the enzyme activity also increased, reaching about 149% of the control after 24 h, and consequently decreased to nearly the control value (115% of the control) in roots exposed to salt for 6 days (Figure 2B,C). Thus, the observed increase in the H_2_O_2_ level occurred in parallel with the enhanced NADPH oxidase activity. To verify whether the observed modulations of enzyme activity were a result of changes at transcriptional level, the *Rboh* gene expression was measured in the roots of seedlings exposed to salt stress. It was demonstrated that two of nine cucumber genes, *CsRbohD* and *CsRbohF1,* were significantly up-regulated by treatment with 100 mM NaCl for 1 h, reaching a 4,5-fold and 2,2-fold increase, respectively, in comparison to the control (Figure 2D). When the plants were exposed to salt for a longer time, the most pronounced effect of 6-day treatment with 50 mM NaCl on *CsRbohH1* expression (a 2,4-fold increase) was observed (Figure 2E).

**Figure 3:**
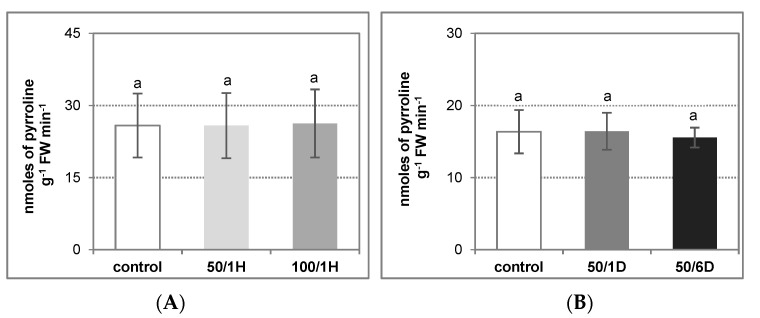
Diamine oxidase activity in the tissues of cucumber roots treated with: (**A**) 50 mM or 100 mM NaCl for 1 h (50/1H and 100/1H, respectively); (**B**) 50 mM NaCl for 1 or 6 days (50/1D and 50/6D, respectively). DAO activity was assayed in the roots, as described in Materials and Methods. The presented results are the means ± SD of three independent experiments, with each experiment performed in triplicate, and are expressed as nmoles of Δ^1^-pyrroline g^−1^ FW min^−1^. Means with the same letter are not significantly different (*p* < 0.05).

Apoplastic diamine oxidase (DAO) and polyamine oxidase (PAO) appeared to be the other enzymes responsible for H_2_O_2_ generation. Our results indicated that DAO activity remained at a similar level in both the control and the salt-stressed plants (Figure 3A,B). On the other hand, in the cucumber roots, the PAO activity was maintained at a very low level, several times lower than the DAO, regardless of the growing conditions (personal communications: Janicka, M., 2021, available in the repository) and, for this reason, it has not been studied. This suggests that the elevation in the H_2_O_2_ amount could be related to the plasma membrane NADPH activity.

**Figure 4:**
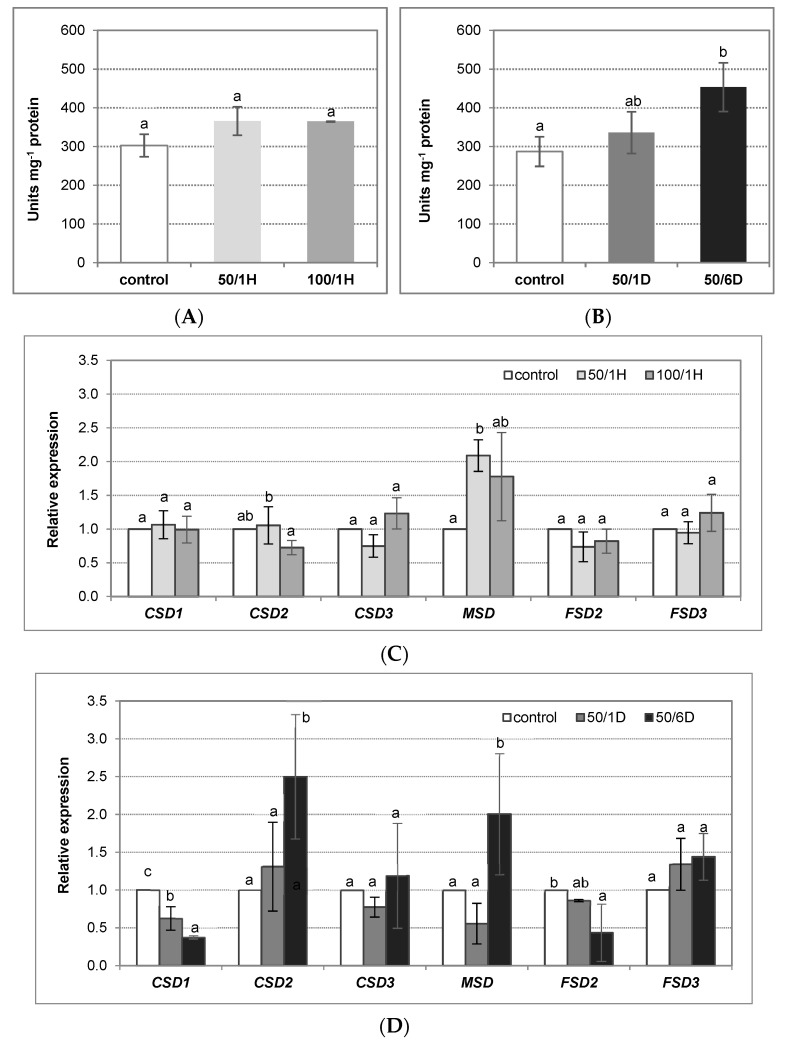
Superoxide dismutase activity (**A**,**B**) and relative expression of SOD encoding genes (**C**,**D**) in the tissues of cucumber roots exposed to salinity. The cucumber seedlings were treated with: (**A**,**C**) 50 mM or 100 mM NaCl for 1 h (50/1H and 100/1H, respectively); (**B**,**D**) 50 mM NaCl for 1 or 6 days (50/1D and 50/6D, respectively). (**A**,**B**) SOD activity was determined in the roots, as described in Materials and Methods. The presented results are the means ± SD of three independent experiments, each run in triplicate, and are expressed as units g^−1^ FW. (**C**,**D**) The total RNA was isolated from the cucumber roots, and real-time PCR analysis was performed, with *CsCACS* as a reference gene, as described in Materials and Methods. The results are shown as the means ± SD of three replications. Different letters show different homogeneous groups of means calculated by Duncan’s multiple range test (significant at *p* < 0.05).

The activity of the plasma membrane NADPH oxidase is related to the action of the superoxide dismutase (SOD). It was found in cucumber that SOD activity was enhanced by salt, similarly to NADPH oxidase. However, a significant increase, by about 60%, was observed in the roots of the plants treated with salt for 6 days (Figure 4A,B). Analysis of six genes encoding SOD in the cucumber genome—including *CsCSD1*, *2*, and *3* belonging to Cu/Zn-SODs, *CsMSD* representing Mn-SODs as well as *CsFSD2* and *3* identified as Fe-SOD—indicated that the expression of one of them, *CsMSD*, was upregulated about twice in seedlings exposed to 50 mM NaCl for both 1 h and 6 days. On the other hand, the transcript level of *CsCSD2* significantly increased (a 2,5-fold increase) only after plant exposure to salt for 6 days (Figure 4C,D).

**Figure 5:**
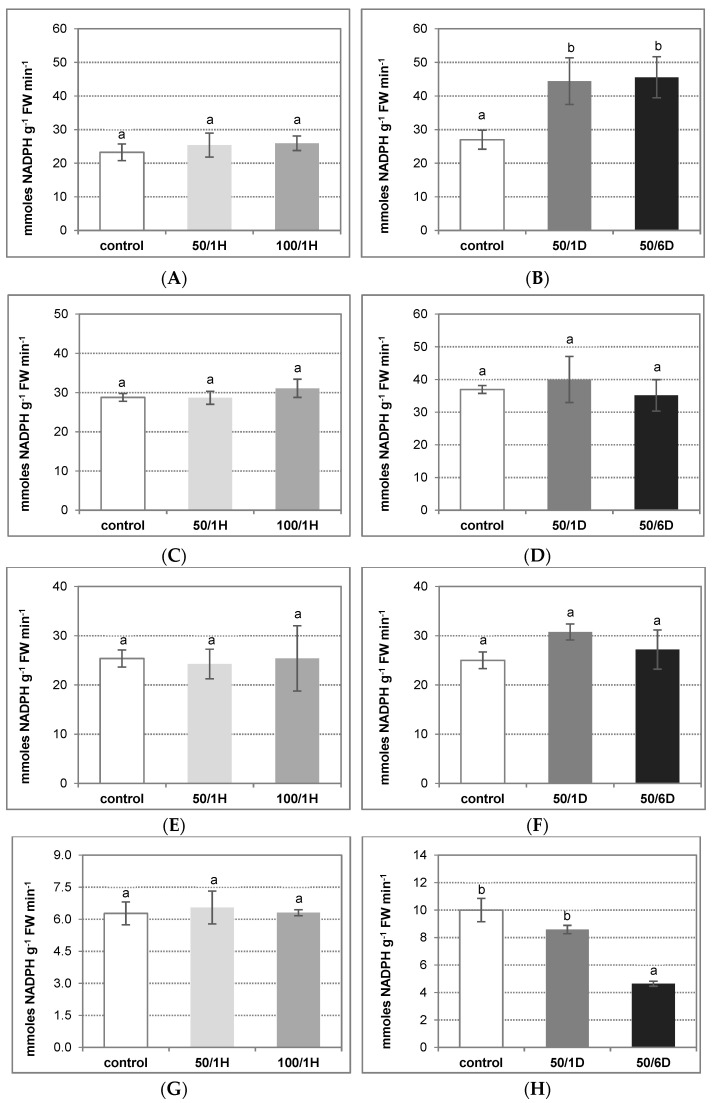
Activity of NADPH-generating enzymes: G6PDH (**A**,**B**), 6PGDH (**C**,**D**), NADP-ICDH (**E**,**F**), and NADP-ME (**G**,**H**) in the roots of cucumber seedlings exposed to salinity. The cucumber seedlings were treated with: (**A**,**C**,**E**,**G**) 50 mM or 100 mM NaCl for 1 h (50/1H and 100/1H, respectively); (**B**,**D**,**F**,**H**) 50 mM NaCl for 1 or 6 days (50/1D and 50/6D, respectively). The activities of NADP dehydrogenases were determined in the roots, as described in Materials and Methods. The presented results are the means ± SD of three independent experiments, each run in triplicate, and are expressed as nmoles of NADPH g^−1^ FW min^−1^. Different letters show different homogeneous groups of means calculated by Duncan’s multiple range test (significant at *p* < 0.05).

The activity of NADPH oxidase can be dependent on NADPH as its metabolic substrate. For this reason, the role of four NADPH-generating enzymes, i.e., glucose-6-phosphate dehydrogenase (G6PDH), 6-phosphogluconate dehydrogenase (6PGDH), NADP-isocitrate dehydrogenase (NADP-ICDH), and NADP-malic enzyme (NADP-ME), was examined in cucumber plants exposed to salinity (Figure 5). Among the enzymes tested, G6PDH was found to be significantly stimulated by salt applied for a longer period. In roots stressed with 50 mM NaCl for 1 and 6 days, the enzyme activity reached about 160–170% of the control value (Figure 5A,B). On the other hand, the activities of both 6PGDH and NADP-ICDH were relatively unaffected by NaCl, achieving similar levels in the roots of control as well as salt-treated plants (Figure 5C–F). In contrast to the above results, NADP-ME was visibly inhibited under longer salinity stress. The observed decrease in its activity was closely related to the time of NaCl treatment, and reached about 50% of the control in roots stressed with salt for 6 days (Figure 5G,H). Thus, it seems very likely that G6PDH is responsible for providing the substrate for NADPH oxidase under salt stress condition.

**Figure 6:**
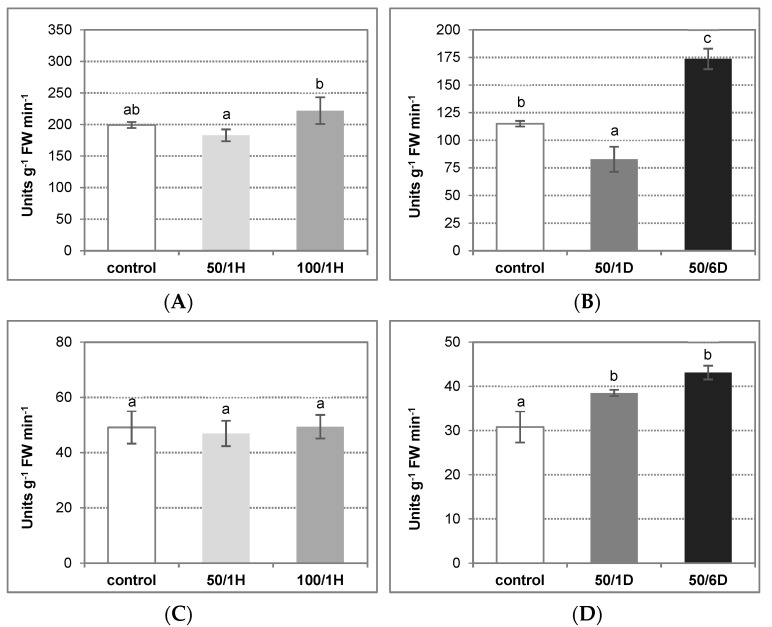
Catalase (**A**,**B**) and ascorbate peroxidase (**C**,**D**) activity in the tissues of cucumber roots exposed to salinity. The cucumber seedlings were treated with: (**A**,**C**) 50 mM or 100 mM NaCl for 1 h (50/1H and 100/1H, respectively); (**B**,**D**) 50 mM NaCl for 1 or 6 days (50/1D and 50/6D, respectively). The activities of antioxidant enzymes were determined in the roots, as described in Materials and Methods. The presented results are the means ± SD of three independent experiments, each run in triplicate, and are expressed as units g^−1^ FW min^−1^. Different letters show different homogeneous groups of means calculated by Duncan’s multiple range test (significant at *p* < 0.05).

To minimize hydrogen peroxide accumulation, plants activate the H_2_O_2_-metabolizing enzymes, including catalase (CAT) and ascorbate peroxidase (APX). In the cucumber root tissues, CAT activity was reduced by about 30% after one day of plant treatment with 50 mM NaCl, as compared to the control plants. Longer plant exposure to this salt concentration (6 days) caused a significant increase in enzyme activity, which achieved above 150% of the control value. CAT activity did not change significantly in the roots treated with salt for 1 h (Figure 6A,B). On the other hand, APX activity was stimulated in the cucumber seedlings stressed with NaCl for a longer time period, reaching its highest level (140% of the control) in roots exposed to salt for 6 days (Figure 6C,D).

**Figure 7:**
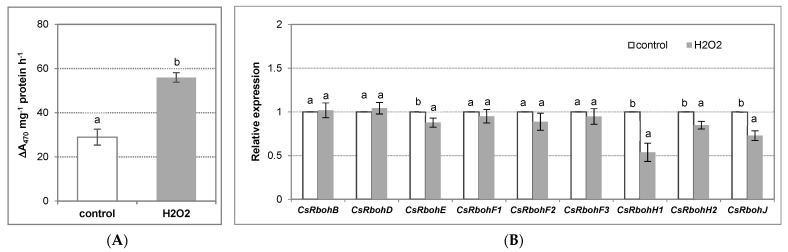
NADPH oxidase catalytic activity (**A**) and relative expression of *CsRboh* genes (**B**) in cucumber roots treated with H_2_O_2_. The cucumber seedlings were treated with 5 mM H_2_O_2_ for 24 h, as described in Materials and Methods. (**A**) The plasma membrane vesicles were isolated from the cucumber roots, as described in Materials and Methods. The presented results are the mean ± SD of three independent experiments, with each experiment performed in triplicate, and are expressed as the ΔA_470_ of formazan XTT mg^−1^ protein h^−1^. (**B**) The total RNA was isolated from the cucumber roots, and real-time PCR analysis was performed, with *CsTIP41* as a reference gene, as described in Materials and Methods. The results are shown as the means ± SD of three replications. Different letters show different homogeneous groups of means calculated by Duncan’s multiple range test (significant at *p* < 0.05).

The possibility that NADPH oxidase can be subjected to regulation by H_2_O_2_ was also studied. The study demonstrated that exogenously applied H_2_O_2_ significantly stimulated the enzyme. The activity of the NADPH oxidase increased 1.9-fold in the roots of seedlings treated with this signaling molecule for 24 h (Figure 7A). Furthermore, it was indicated that the H_2_O_2_-induced increase in the enzyme activity was not related to the activation of *CsRboh* gene expression in roots (Figure 7B).

## 4. Discussion

To survive in soils contaminated with high salt concentration, plants have developed different adaptive mechanisms, in which signaling molecules seem to play an essential role. Many studies have indicated that, in response to salinity, enhanced levels of ROS, including hydrogen peroxide, are generated in plant tissues [30]. Consequently, oxidative damage of cellular components occurs, affecting plant cell functioning [30]. On the other hand, an increase in ROS content detected after exposure to salt can act as an early component of salinity sensing (acclimation signal), activating downstream H_2_O_2_-dependent signaling pathways, and leading to stress responses [9]. Among others, ROS were found to activate the MAPK cascade and potassium channels, and to modulate ion fluxes in the cells [9]. For this reason, it has been suggested that the coordinated ROS generating and scavenging system plays a crucial role in plant adaptation to salt stress [31].

Although knowledge about salinity sensing by plant cells is still limited, several sensory mechanisms have been proposed [32]. Moreover, it is supposed that different systems can operate in the same cell and at the same time, including the plasma membrane NADPH oxidase [33]. Accordingly, it has been shown in several plant species that salt stress rapidly induces a transient increase in NADPH oxidase activity, and up-regulates the expression of *Rboh* genes. This increase in enzyme activity is accompanied by H_2_O_2_ accumulation [34,35]. *Arabidopsis* mutants with defect in functional RBOH isoforms (AtrbohD and AtrbohF) exhibit increased Na^+^ hypersensitivity, and lack of ROS accumulation in the xylem vessels [12,36]. Thus, it has been proposed that RBOH could be involved in the control of sodium loading to the xylem, protecting leaves from the adverse effects of salinity [37].

In contrast to the above results, NaCl can also negatively affect the NADPH oxidase. Srivastava, et al. [38], have demonstrated that enzyme activity decreases in a glycophyte *Brassica juncea* (low salt accumulator), and that it remains unaffected in a halophyte *Sesuvium portulacastrum* (high salt accumulator) under salt stress conditions. Decrease in the ROS level related to inhibited RBOH activity has been shown in maize seedlings treated with NaCl [39]. Similarly, in sugar beet, H_2_O_2_ content was significantly lower in salt-treated tissues compared to the controls. This correlated with decreased NADPH oxidase activity and down-regulation of *RBOH* gene transcription [40].

Our results indicate that an enhanced H_2_O_2_ level occurred in cucumber roots stressed with 100 mM NaCl for 1 h and 50 mM NaCl for 1 day. At the same time, NADPH oxidase activity was significantly stimulated. The observed increase in enzyme activity was related to the enhanced expression of two *Rboh* genes, *CsRbohD* and *CsRbohF1*. Similarly, Niu, et al. [33], using 75 mM NaCl concentration, indicated that the H_2_O_2_ content is rapidly elevated in the roots of salt-treated pumpkin-grafted cucumber plants, reaching a peak at 3 h and decreasing during the 3–12 h period. This increase is related to the NaCl-induced NADPH oxidase activity, as well as enhanced expression of *RbohD* and *RbohF* transcription level.

The generation of ROS in the apoplastic space could be related not only to the activation of the cell membrane-bound RBOH but also the apoplastic polyamine or diamine oxidases [41]. In salt-stressed maize leaves, RBOH activity is inhibited. Under such condition, the apoplastic polyamine oxidase, which activity increases, is responsible for the elevation in H_2_O_2_ content required for cell wall loosening and leaf growth [42]. In our study, DAO activity remained unchanged in the cucumber seedlings, regardless of the salt concentration and treatment time. Furthermore, PAO activity maintained a very low level in the cucumber roots (personal communications: Janicka M, 2021, available in the repository). This may suggest that RBOH participates in H_2_O_2_ generation in the apoplast in cucumber roots under salt stress. As we demonstrated earlier, the H_2_O_2_ content only slightly increased in 1-day stressed roots, whereas it was very high in 6-day treated roots of *Cucumis sativus* var. Wisconsin under salinity condition [43]. It is interesting that such visible differences occurred in two cultivars of the same plant species, suggesting some distinct stress responses. Duan, et al. [44], using two cucumber cultivars with different salt tolerance—Changchun mici (tolerant) and Jinchun No. 2 (more sensitive)—exposed to salinity for 7 days, demonstrated that 50 mM NaCl caused a significant increase in DAO activity in the roots of the former, but only a slight increase (only observed after 3 days of stress) in the roots of the latter. Similar to our results, salt treatment had no effect on DAO activity in the roots of rice seedlings [45]. The authors concluded that DAO does not contribute to H_2_O_2_ production in the cell wall of stressed plants. In contrast, salt stress was found to both strongly promote DAO activity and to stimulate polyamine degradation in the root tissues of maize seedlings [46] and lupine seedlings [47]. Thus, it seems obvious that different mechanisms function in plant cells to enhance H_2_O_2_ production, depending on the plant species/cultivar and its sensitivity to salt as well as treatment conditions.

The activity of plasma membrane NADPH oxidase can be regulated in several ways [2]. Research using *Arabidopsis* has revealed some regulatory mechanisms of RBOH proteins, which depend on different signaling effectors. Thus, it has been suggested that differences in regulatory mechanisms may be responsible for diverse RBOH functions in plant cells [48]. A few studies have shown that, under salinity conditions, cytosolic Na^+^ accumulation followed by Ca^2+^ elevation can lead to the activation of RBOH and, consequently, to the accumulation of apoplastic H_2_O_2_ [37]. In our research, treatment of plants with H_2_O_2_ clearly activated NADPH oxidase, indicating that this enzyme remains under feedback control. However, the observed effect did not include the gene expression level, suggesting some post-translational regulatory mechanisms or additional signaling events, such as nitrosylation [49], H_2_S-dependent persulfidation [50], and reversible phosphorylation [51].

Substrate availability appears to be another factor involved in controlling NADPH oxidase activity. Several enzymes are responsible for NADPH delivery function in plant cells [52]. It has been proposed that various NADPH-generating dehydrogenases could provide this essential cofactor for different NADPH-dependent processes. Moreover, there is evidence that one or more NADP dehydrogenases are regulated under specific stress conditions [52]. In *Arabidopsis* seedlings, the NADP-ICDH, which activity increased, appeared to act as a barrier in the defense mechanisms activated against salt stress [53]. In contrast, a decrease in the activities of the main NADPH-generating enzymes, especially NADP-ICDH, has been demonstrated in tomato roots under salinity conditions [54]. Among the NADP dehydrogenases (G6PDH, 6PGDH, NADP-ME, and NADP-ICDH) analyzed in the present work, the NADP-ICDH activity remained relatively unaffected, whereas the G6PDH activity was importantly enhanced in the roots of 1-week-old cucumber seedlings treated with NaCl for 1 and 6 days, suggesting that this protein may serve as a source of NADPH for RBOH under stress conditions. On the other hand, treatment of 5-week-old cucumber plants with 100 mM NaCl for 1–3 days significantly reduced G6PDH activity in roots, but stimulated it in leaves [55]. As it was reported in *A. thaliana* exposed to high salinity, the cytosolic G6PDH isozyme (G6PD6) was subjected to phosphorylation, and this modification was responsible for the observed enzyme activation [56]. Studies using *Arabidopsis* mutants revealed that both the G6PD enzymatic activity and the expression of *G6PD5* and *G6PD6* decreased in *atrbohD1*, *atrbohF1*, and *atrbohD1/F1* mutants in comparison to the WT plants, confirming the involvement of cytosolic G6PDH in RBOH-dependent ROS generation [57]. Scharte, et al. [58], have proposed that cytosolic G6PDH functions as an essential element of plant defense reactions, and that its modulation is important to enhance stress tolerance.

Plasma membrane-localized NADPH oxidases are a major source of cellular O_2_^−^ radicals, which are transformed by superoxide dismutases to produce less reactive H_2_O_2_. Related to the metal cofactor at the active center, three distinct isoenzymes function in plant cells: Mn-SODs are present in the mitochondria and peroxisomes; Cu/Zn-SODs mainly in the cytosol, mitochondria, and plastids; while Fe-SODs are localized in the cytosol, mitochondria, chloroplasts, and peroxisomes [59]. Many studies have confirmed that salt stress triggers an increase in total SOD activity, especially in the leaves of plants treated with high doses of NaCl for several days [59]. In cucumber root cells, total SOD activity as well as *CsMSD* and *CsCSD2* expression increased significantly in the roots of seedlings exposed to 50 mM NaCl for 6 days, when NADPH oxidase activity and H_2_O_2_ level were lowered to nearly control values. This suggests that hydrogen peroxide generated by SOD may be effectively decomposed by H_2_O_2_-metabolizing systems. Hossain, et al. [40], have shown that in a highly salt-tolerant sugar beet stressed with 300 mM NaCl for up to 14 days, H_2_O_2_ content is always significantly lower in the salt-treated leaves in comparison to the control. This was correlated with the suppressed NADPH oxidase activity and gene expression (with the exception of *RBOHB*), as well as with the up-regulation of SOD activity and increased transcription of genes encoding this enzyme (with the exception of *FeSOD1*). Such a system, together with enhanced expression of genes for alternative oxidase and plastid terminal oxidase, is responsible for maintaining the low ROS level under severe salinity [40].

It is assumed that early production of H_2_O_2_ acts as a signal to induce antioxidant systems and prevent subsequent ROS generation and ROS-dependent cell damage [37]. In *Arabidopsis* under short-term salt treatments, RbohD and RbohF were suggested to be required in this process [13]. The main enzymes able to break down H_2_O_2_ are catalase, with the highest turnover rates, and ascorbate peroxidase, exhibiting higher affinity for H_2_O_2_ than CAT [60,61]. In accordance with the above assumption, in this study, both the up-regulation of APX and CAT activity were shown in the roots of cucumber seedlings exposed to salinity for 6 days, confirming the involvement of both enzymes in maintaining the low H_2_O_2_ level under prolonged salt exposure. In contrast, catalase activity was inhibited in roots treated with salt for 1 day. Stimulation of both APX and CAT in response to NaCl has been reported in many plant species. It has been shown to be associated with decreased oxidative damage and improved tolerance to salinity. Similar to SOD, a greater increase in CAT activity was observed in salt-tolerant plants/genotypes than in more sensitive ones [60]. Furthermore, in some glycophytes, including rice, the activity of this enzyme decreased after NaCl treatment [62].

## 5. Conclusions

At the early stages of salt stress (1 h and 1-day-treatment), the plasma membrane NADPH oxidase was activated in the cucumber (*Cucumis sativus* var. Krak) seedlings. This activation correlated with an observed enhancement in H_2_O_2_ content, suggesting the involvement of NADPH oxidase in the generation of H_2_O_2_, which may function as a signaling molecule participating in stress response. The observed stimulation of NADPH oxidase may have been at least partially related to the induction of some *CsRboh* genes, as well as the increased activity of G6PDH, providing NADPH. Another factor which may contribute to positive RBOH regulation at the post-translational level, could be H_2_O_2_. This proposal, however, requires additional studies concerning direct or indirect H_2_O_2_ action.

After 6-day exposure to salt stress, the plasma membrane NADPH oxidase activity, and consequently the H_2_O_2_ level, were diminished. This protected the cells against the toxic effect of high H_2_O_2_ concentrations. Additionally, antioxidant enzymes, including SOD, APX, and CAT, were significantly activated to maintain low H_2_O_2_ content in cells, and to avoid ROS-dependent damages. However, to explain the precise mechanism of NADH oxidase regulation, and its interrelation with the antioxidant system in cucumber seedlings subjected to salinity, further analysis is needed.

## Figures and Tables

**Table 1:** The list of primers used in qRT-PCR reactions.

**Gene**	**Forward Primer**	**Reverse Primer**
*CsTIP41*	CAACAGGTGATATTGGATTATGATTATAC	GCCAGCTCATCCTCATATAAG
*CsRbohB*	AGAGTCGGCTTCAGCGG	TCTCCTCCTCTGATGTTCTGAACGG
*CsRbohD*	TCTTCTTCTTCTTCCTCCCTCAAAGCC	GAAAGTTCAGGGTCTTCAAGAGAGTTG
*CsRbohE*	TCACTTTGGAAGTTGAGGATGATTCTGTT	GTCGAGAGAGGAGGCAGTGG
*CsRbohF1*	GCTTCGATTTCGAGATCGCCGAC	AAATCCATTTCCACCACCAGAAGAATGT
*CsRbohF2*	TCGCATCGGTTTCGGCAG	AGTTCACGGTTGAAGATGACGC
*CsRbohF3*	AAGAGAAGTCCGAGAATGAGGTTTAT	CTCCTGAGATTCTGACATTCCAATGC
*CsRbohH1*	AGATTCCGATGTGATAGATGTCATGGT	TTGGACTGTCTTCTTCCAAGCCTC
*CsRbohH2*	AAATCCAGGAGAATGCCCAC	TGAGAAGTCTCAGCGACGG
*CsRbohJ*	CTGACGATGGGATTACTCTGCAACA	GAAGCAGTCCACTTCAGTATGGTCT
*CsCACS*	TGGGAAGATTCTTATGAAGTGC	CTCGTCAAATTTACACATTGGT
*CsCSD1*	AAAGATGGTGAAGGCTGTGG	CATGTTGTTTTCCAGCAG
*CsCSD2*	CCCATTCTCCAATTCTTCAT	TTGGGTGAGCGTGACAA
*CsCSD3*	CCAAAAGAGGGGGAATTTTT	GCCTCTGACGTTGGAATCTC
*CsMSD*	CGCTTCGAATTCTAGGCAGG	CCTTTGTTGATGGCCTCGTG
*CsFSD2*	TAACACGGTTGACCATCC	TTCTGTAGCCTCAAGTCT
*CsFSD3*	TCTGATGGAACCACAAGAG	TGTGGCCATATAGAATATCATTT

## Data Availability

The data presented are available in this manuscript and in the repository at https://www.repozytorium.uni.wroc.pl/dlibra/publication/139358/edition/128721# (accessed on 10 September 2021).
